# RMP predicts survival and adjuvant TACE response in hepatocellular carcinoma

**DOI:** 10.18632/oncotarget.3092

**Published:** 2014-12-30

**Authors:** Jian Zhang, Tian-Yi Jiang, Bei-Ge Jiang, Chun Yang, Ye-Xiong Tan, Ning Yang, Yu-Fei Pan, Zhi-Wen Ding, Guang-Zhen Yang, Meng-Chao Wu, Li-Wei Dong, Hong-Yang Wang

**Affiliations:** ^1^ International Cooperation Laboratory on Signal Transduction, Eastern Hepatobiliary Surgery Institute, The Second Military Medical University, Shanghai, P. R. China; ^2^ National Center for Liver Cancer, Shanghai, P.R. China; ^3^ Department of Surgery, Eastern Hepatobiliary Surgery Hospital, The Second Military Medical University, Shanghai, P.R. China; ^4^ State Key Laboratory of Oncogenes and related Genes, Shanghai Cancer Institute, Renji Hospital, Shanghai Jiaotong University School of Medicine, P.R. China; ^5^ Current address: Department of Cardiac Surgery, General Hospital of Shenyang Military Area Command, Shenyang, P. R. China

**Keywords:** hepatocellular carcinoma, RPB5-mediating protein, adjuvant transcatheter arterial chemoembolization, overall survival, prognosis

## Abstract

Adjuvant transcatheter arterial chemoembolization (TACE) protects against hepatocellular carcinoma (HCC) and is associated with reduced disease recurrence and improved outcome after surgery. However, deterioration of liver function after TACE negatively impacts the patient prognosis and limits it use as an option to prolong survival. We analyzed two independent cohorts that included a total of 510 patients with HCC who had undergone tumor resection. Immunohistochemistry assay was used to measure RPB5-mediating protein (RMP) expression and assessed their association with recurrence rate and response to therapy with adjuvant TACE. In patients with HCC, the expression of RMP in tumor is associated with age, gender, tumor size, portal venous invasion, TNM stages, BCLC stages and overall survival. Among patients with high RMP expression, adjuvant TACE after resection was associated with early recurrence. Even in the patients with small tumor size (no more than 5 cm) or no venous invasion, RMP status is associated with response to adjuvant TACE. RMP status in tumors may be a useful marker in estimating prognosis in patients with HCC and in assisting in the selection of patients who are likely to benefit from adjuvant TACE to prevent relapse.

## INTRODUCTION

Hepatocellular carcinoma is the fifth most common cancer worldwide and is blamed for nearly 500,000 deaths each year [1]. The primary goal of therapy for HCC is to improve long-term survival in patients amenable to surgical therapy and possibly affecting cure, whether it is via resection or transplantation. Nonsurgical therapies such as radiofrequency ablation (RFA), cryoablation, microwave coagulation, percutaneous ethanol injection (PEI), and TACE have traditionally been used for local tumor control [2]. For lacking of donor liver, hepatectomy remain the first option for patients who have the optimal profile [3]. Recurrence of HCC following resection is clearly a common occurrence, with 50% to 80% of patients experiencing recurrence within 5 years after resection, and the majority within 2 years [4, 5]. The mechanism is typically not an inadequate resection, but rather de novo tumor formation in the cirrhotic liver, or intrahepatic metastases that were too small to be detected/identified at the time of resection[2].

To inhibit remnant tumor growth, early detect and treat tiny metastases, TACE is one of the most commonly used adjuvant managements for preventing recurrence and prolonging the survival of patients postoperatively [6, 7]. The patients with large tumors (more than 5cm), venous invasion or intrahepatic metastases are recommended to receive TACE 1-2 months after resection [8-10]. However, deterioration of liver function after TACE may negatively impact the patient prognosis and liver function [11]. Exception of histological characters, detailed analysis and characterization of the molecular mechanisms and subsequently individual prediction of corresponding prognostic traits would revolutionize treatment of HCC and is the key goal of modern personalized medicine [12].

RMP, also known as unconventional prefoldin RPB5 interactor (URI), is associated with the RNA polymerase II subunit, RPB5, and coordinates gene expression [13-15]. RMP is recognized as oncoprotein in ovarian cancer and HCC [16-19]. We found that RMP promoted HCC cells metastases and was associated with portal vein tumor thrombosis (PVTT) formation in HCC, which markedly deteriorated hepatic function and predicted poor survival [20]. Therefore we hypothesized that the effect of postoperative adjuvant TACE on survival among patients with high RMP expression tumors might differ from the effect among those with low RMP expression tumors. In this study we investigated the prognostic value of RMP and its response to postoperative adjuvant TACE in HCC patients.

## RESULTS

### Patient Characteristics

Patient characteristics of both cohorts are shown in Table 1. All the patients were underwent surgery with or without adjuvant TACE after surgery and diagnosed by radiologic imaging plus pathology. The patients in the training cohorts were subdivided into two groups according to RMP immunostaining intensities (Fig. S1A). Similar observations were obtained when the two different sets were analyzed separately or when the pooled cohorts were evaluated by three different observers. In the training cohort (n = 263), most of the patients were men (90%, n=236), were long-term carriers of hepatitis B virus (HBV) (86%, n=227), and an elevated serum level of AFP (alpha-fetoprotein) (65%, n=171); 87% of the patients (n=230) had a single tumor nodule and 30% of the patients (n=78) had venous invasion at the time of resection. 46% of patients (n=122) received adjuvant TACE after the surgery within 1-2 months, while 54% patients (n=141) followed up only at the first two months after resection. In the validation cohort (n=247), 151 patients (55%) were received TACE after liver resection (Table 1).

**Table 1:** Characteristics of Patients in the Training and Validation Cohort

		Training cohort			Validation cohort	
		N	%		N	%
Age (years)		263	100		247	100
	Mean, SD	50.1, 10.50	-		50.17 10.7	
	Range	22-77	-		26-77	
Sex	Male	236	90		213	86
	Female	27	10		34	14
HBs Ag	Negative	36	14		35	14
	Positive	227	86		212	86
Serum AFP	≤400 (ng/ml)	92	35		86	35
	>400 (ng/ml)	171	65		161	65
Largest tumor size	≤5 (cm)	114	43		82	33
	>5 (cm)	149	57		165	67
Tumor number	Single	230	87		209	85
	Multiple	33	13		38	15
Venous invasion	Negative	185	70		158	64
	Positive	78	30		89	36
BCLC stage	A	107	40		75	30
	B	78	30		83	34
	C	78	30		89	36
TNM	I/ II	152	58		126	51
	III/IV	111	42		121	49
Adjuvant TACE	Yes	122	46		135	55
	No	141	54		112	45

### RMP Expression Predicts Poor Prognosis of HCC

Of the training cohort patients, intriguingly, RMP expression levels were found to be significantly associated with age of the patients (P=0.027), gender of the patients (P=0.040), tumor size (P=0.015), portal venous invasion (P=0.001), TNM stages (p=0.001) and BCLC stages (p=0.004) in training set (Table 2), and similar results were observed in the validation cohort (Table S1).

**Table 2:** Relationship between RMP protein expression and clinicopathologic characteristics in training set (n=263)

Characteristics	No. patients	RMP expression in HCC		P value
		Low	High	
Age (yrs)				0.027
≤49	70	31	39	
>49	193	115	78	
Gender				0.040
Male	236	126	110	
Female	27	20	7	
HBs Ag				0.714
Negative	36	21	15	
Positive	227	125	102	
Serum AFP				0.780
≤400(ng/ml)	92	50	42	
>400(ng/ml)	171	96	75	
Largest tumor size				0.015
≤5 (cm)	114	73	41	
>5 (cm)	149	73	76	
Tumor number				0.168
Single	230	124	106	
Multiple	33	22	11	
Venous invasion				0.001
Negative	185	115	70	
Positive	78	31	47	
BCLC stage				0.004
A	107	67	40	
B	78	48	30	
C	78	31	47	
TNM				0.001
I/ II	152	98	54	
III/IV	111	48	63	

**Figure 1:**
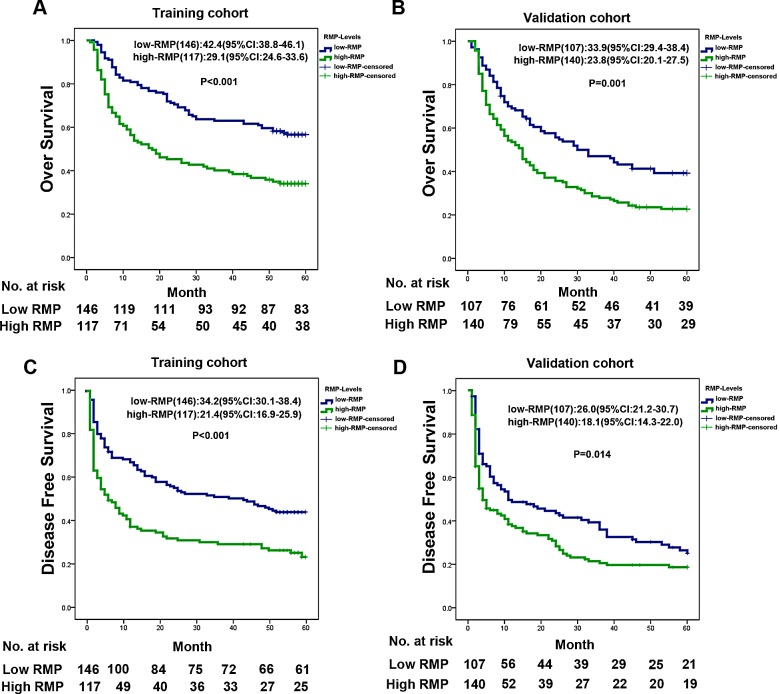
RMP expression associated with overall survival (A, B) Kaplan-Meier analysis of the correlation between RMP expression level and overall survival of HCC patients in training cohort (A) and validation cohort (B); (C, D) Kaplan-Meier analysis of the correlation between RMP expression level and disease free survival of HCC patients in training cohort (C) and validation cohort (D).

We evaluated the correlation between RMP levels and patient outcomes by a regression spline analysis. Kaplan–Meier analysis revealed that the high-expressed RMP patients had a significantly shorter OS than the low-RMP patients in training cohorts (OS high-RMP versus low-RMP: 29.1 versus 42.4 months [95% confidence interval, CI: 24.6-33.6 versus 38.8-46.1], P<0.001) (Fig. 1A). We tested the reproducibility of the findings by another validation cohort and found that high RMP levels were associated with poor OS (median OS for high-RMP versus low-RMP: 23.8 versus 33.9 months; 95% CI: 29.4-38.4 versus 20.7-27.5, P=0.001, Fig. 1B). Furthermore, high-expressed RMP predicted recurrence of HCC in training cohort (median DFS for high-RMP versus low-RMP: 21.4 versus 34.2 months; 95% CI: 16.9-25.9 versus30.1-38.4, P<0.001, Fig. 1C) and validation cohort (median DFS for high-RMP versus low-RMP: 18.1 versus 26.0 months; 95% CI: 14.3-22.0 versus 21.2-30.7, P=0.014, Fig. 1D).

### Prognostic Significance of Postoperative Adjuvant TACE within the RMP Level

**Figure 2:**
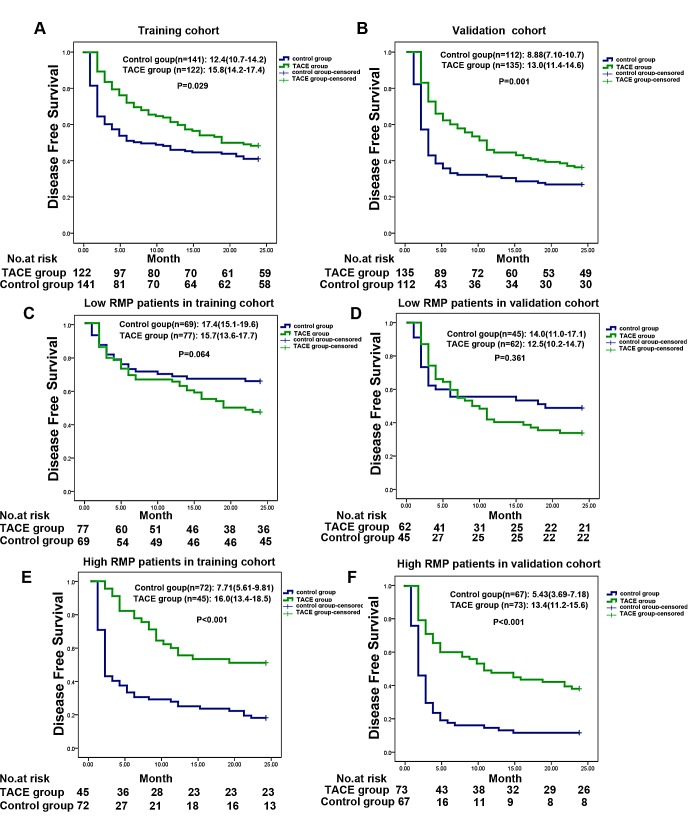
Prognostic significance of postoperative adjuvant TACE within the RMP level (A,B) Kaplan-Meier analysis of the correlation between adjuvant TACE therapy and 2 years disease free survival in training cohort (A) and validation cohort (B). (C,D) Kaplan-Meier analysis of the correlation between adjuvant TACE therapy and 2 years disease free survival in patients with low RMP expression in training cohort (C) and validation cohort (D). (E,F) Kaplan-Meier analysis of the correlation between adjuvant TACE therapy and 2 years disease free survival in patients with high RMP expression in training cohort (E) and validation cohort (F).

Adjuvant TACE was one of the most used methods to prevent tumor recurrence. In our two cohorts, adjuvant TACE prolonged the OS (Fig. S2A, B). However, adjuvant TACE has little effect on 5 years disease free survival (DFS) (Fig. S2C, D). As cumulative recurrence rate curves shown in Fig.S2C and 2D, early tumor recurrence has been decreased in both cohorts, which indicated that the function of adjuvant TACE was to inhibit remnant tumor growth. Therefore, 2 years DFS affected by adjuvant was compared in two cohorts and the results showed that TACE inhibited recurrence of HCC in training cohort (median DFS for adjuvant TACE group versus control group: 15.8 versus 12.4 months; 95% CI: 14.2-17.4 versus 10.7-14.2, P=0.029, Fig. 2A). The similar results were observed in validation cohort (median DFS for adjuvant TACE group versus control group: 13.0 versus 8.88 months; 95% CI: 11.4-14.6 versus 7.10-10.7, P=0.001, Fig. 2B).

Because RMP may predict early metastasis and recurrence, we next evaluated whether RMP expression in the tumor was associated with the response of patients to adjuvant TACE therapy. Patients with low RMP expression in tumors had no significant improvement in early recurrence rate after receiving adjuvant therapy with postoperative TACE, as compared with those without adjuvant TACE in both training and validating cohorts (Fig. 2C,D). In contrast, patients with high RMP expression in tumors had a better response to adjuvant TACE (median DFS [95% CI] for adjuvant TACE [n=45] versus control [n=72]: 16.0 [13.4-18.5] versus 7.71[5.61-9.81] months, P<0.001, Fig. 2E). This finding was validated in validation cohort (median DFS [95% CI] for adjuvant TACE [n=73] versus control [n=67]:13.4 [11.2-15.6] versus 5.43 [3.69-7.18] months, P<0.001, Fig. 2F). As the early recurrence is one of the main factors for overall survival of HCC patients, we compared the effect of adjuvant TACE on overall survival in different RMP-expressed patients (Fig.S3A-D). Adjuvant TACE was not a good option to prolong overall survival of the patients with low RMP expression (Fig.S3A,B); while, in patients with high RMP expression, TACE was an effective way to improve long term survival (Fig.S3C,D). In order to avoid the patients selecting bias, we excluded the patients who were bad performance status and poor liver function after resection. The similar tendency was observed (Fig.S4A-F and Fig.S5A-D).

### Uni- and Multivariate Analysis of Prognostic Factors

We used Cox proportional-hazards regression to further evaluate the association between RMP expression and response to adjuvant TACE in both cohorts. In the high RMP-expressed patients of training cohort, adjuvant TACE, age, HBV infection, size of tumor, venous invasion and BCLC stages were significantly associated with tumor recurrence (Table 3). Multivariate Cox regression analysis revealed that adjuvant TACE was an independent prognostic indicator for 2 years DFS in the patients with high RMP expressed of training cohort (hazard ratio [95% CI], 0.473[0.293-0.765], P=0.002) (Table 3). In the validation cohort, Multivariate Cox regression analysis remained reveal that adjuvant TACE was an independent prognostic indicator for 2 years DFS in high RMP expressed patients (hazard ratio [95% CI], 0.434 [0.291-0.646], P=0.002) (Table S2).

**Table 3:** Univariate and multivariate Cox regression analyses of 2 years DFS in different RMP expression patients of the training cohort

Variables	Low-RMP		High-RMP	
	Hazard ration (95% CI)*	p Value	Hazard ration (95% CI)*	p Value
Univariate analysis				
adjuvant TACE (yes vs no)	1.586(0.958-2.627)	0.073	0.382 (0.240-0.606)	**0.000**
Age (>49 years vs ≤49 years)	0.260 (0.156-0.432)	**0.000**	0.401(0.243-0.661)	**0.000**
Gender (male vs female)	0.682(0.311-1.494)	0.682	0.522(0.164-1.658)	0.270
HBs Ag (negative vs positive)	3.261 (1.183-8.985)	**0.022**	2.758(1.112-6.841)	**0.029**
Serum AFP (>400 ng/ml vs≤400 ng/ml)	1.925 (1.094-3.387)	**0.023**	1.600 (0.986-2.596)	0.057
Largest tumor size (>5 cm vs ≤5 cm)	4.058(2.327-7.077)	**0.008**	4.189 (2.327-7.542)	**0.000**
Tumor number(single vs multiple)	2.193(1.227-3.917)	**0.008**	1.433(0.715-2.874)	0.311
Venous invasion (negative vs positive)	2.887 (1.721-4.845)	**0.000**	5.585(3.389-9.205)	**0.000**
BCLC stage (A vs B vs C)	2.369(1.743-3.220)	**0.000**	2.932(2.116-4.062)	**0.000**
TNM (I+II vs III+IV)	4.100(2.499-6.726)	**0.000**	1.137(0.548-2.355)	0.731
Multivariate analysis				
adjuvant TACE (yes vs no)	NA		0.473(0.293-0.765)	**0.002**
Age (>49 years vs ≤49 years)	NA		NA	
HBs Ag (negative vs positive)	4.831(1.739-13.421)	**0.003**	NA	
Serum AFP (>400 ng/ml vs≤400 ng/ml)	NA		NA	
Largest tumor size (>5 cm vs ≤5 cm)	3.294(1.800-6.030)	**0.000**	2.678(1.446-4.959)	**0.002**
Venous invasion (negative vs positive)	NA		3.827(2.262-6.475)	**0.000**
BCLC stage (A vs B vs C)	NA		NA	
TNM (I+II vs III+IV)	3.176(1.855-5.437)	**0.000**	NA	

Univariate analysis, Cox proportional hazards regression; Multivariate analysis, Cox proportional hazards regression; Variables were adopted in multivariate analysis for their prognostic significance by univariate analysis.

### RMP Predicts Response to Adjuvant TACE in Clinical Subgroups

**Figure 3:**
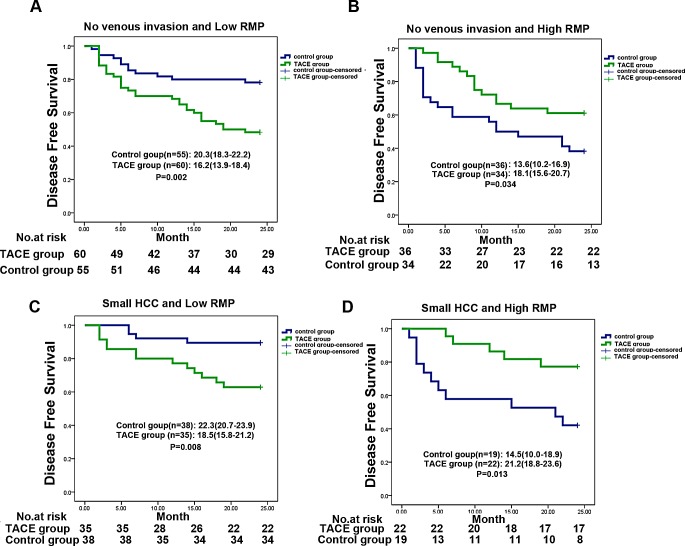
RMP predicts response to postoperative TACE in several clinical subgroups (A,B) Kaplan-Meier analysis of the correlation between adjuvant TACE therapy and 2 years disease free survival in patients with no venous invasion at different RMP expression in training cohort, low RMP(A) and high RMP (B). (C,D) Kaplan-Meier analysis of the correlation between adjuvant TACE therapy and 2 years disease free survival in patients with small HCC at different RMP expression in training cohort, low RMP(C) and high RMP (D).

The HCC patients with large tumors (more than 5 cm) or venous invasion were suggested to receive adjuvant TACE after resection. In our training and validation cohorts, we also found that patients with no venous invasion or small tumor size have a bad response to adjuvant TACE (Fig.S6A, B). Given the independent prognostic significance of RMP levels in the training and the validation cohort, we evaluated the discriminative power of elevated RMP levels within the tumor size or venous invasion status. In patients with no venous invasion of training cohort (n=185), low-expressed RMP patients had a bad response on adjuvant TACE (median DFS [95% CI] for adjuvant TACE [n=60] versus control [n =55]: 16.2 [13.9-18.4] versus 20.3 [19.3-22.2] months, P =0.002, Fig. 3A). On the contrary, of the patients with high-RMP expression, adjuvant TACE group had a better median DFS than control group (median OS [95% CI] for adjuvant TACE [n=34] versus control [n=36]: 19.1 [15.6-20.7] versus 13.6 [10.2-16.9] months; P=0.034, Fig. 3B). A similar trend was found in validation cohort (Fig. S6C, D). In the patients of training cohort with small HCC (n=114), low-expressed RMP patients had a bad response on adjuvant TACE (median DFS [95% CI] for adjuvant TACE [n=35] versus control [n =38]: 18.5 [15.8-21.2] versus 22.3 [20.7-23.9] months, P =0.008, Fig. 3C). Contrarily, adjuvant TACE was associated with significant improvement in 2 years DFS of HCC patients with high RMP expression (median DFS [95% CI] for adjuvant TACE [n=22] versus control [n=19]:21.2 [18.8-23.6] versus 14.5 [10.0-18.9] months, P=0.013, Fig. 3D). Further, the relationship between RMP expression and response of adjuvant TACE in early HCC patients was also test in validation cohort and similar results were observed (Fig.S6E-H).

## DISCUSSION

The frequent postoperative recurrence was a main problem for long survival after resection of HCC and intrahepatic metastases was thought to have a close relation with the postoperative recurrence [21]. There are two recurrence peak of HCC [8]. The early recurrence peak may result from metastasis of primary tumor and adjuvant TACE was one way to inhibit residual tumor. Many evidences suggested that the benefits of adjuvant TACE depended on the selection of patients [9]. In patients who had high risks of residual tumor in remnant liver, adjuvant TACE could improve their survival due to therapeutic actions on the residual tumor [6, 22].

Our analyses revealed that RMP expression was an independent predictor of survival. However, when outcomes of therapy with adjuvant TACE were stratified, only patients whose tumors had high-RMP expression had a favorable response to adjuvant TACE in two independent trials. In our clinical practice, patients with risk factors such as recurrence-large tumor size or venous invasion were suggested to receive adjuvant TACE [6, 22-24]. However, some groups found that adjuvant TACE after hepatectomy demonstrated completely different results [25-28]. Incorporating molecular analysis of primary tumors and recurrences may improve understanding of prognostic factors [28]. Our results showed that even in the patients with low risk factors, small tumor size or venous invasion free, adjuvant TACE was recommended in high RMP expression patients. These results indicate that RMP status in tumors may be a useful tool in estimating prognosis in patients with HCC and in assisting in the selection of patients who are likely to benefit from adjuvant therapy with adjuvant TACE to prevent relapse. In an evaluation of the effects of adjuvant TACE therapy after liver resection prospective studies will be necessary to determine whether adjuvant TACE might be used as a first-line therapy for patients with HCC who have undergone resection and who have tumors with high-RMP expression.

The mechanisms behind the sensitivity of tumors with high-RMP expression to therapy with postoperative TACE are unclear. Immunohistochemistry results suggested that RMP, as a mediator of translation, not only expressed in the nuclear, but also in the cytoplasm. Moreover, Other studies showed that RMP was reported that located on the mitochondrial, which moderated cell apoptosis [16, 17]. Our previous study found that RMP was associated with high metastasis capability by positive regulating IL-6 transcription. Moreover, clinic-pathological parameters and early recurrence analysis both suggested that RMP predicted early metastasis of HCC. Consistently, the patients with high-RMP expression may be metastases at an early stage. One of the main mechanisms of adjuvant TACE to inhibit remnant tumor growth is to kill the early metastasis tumor cells in remnant liver. However, it is difficulty to detect the minimal intrahepatic metastasis before or during operation and the existence of these minimal intrahepatic metastases contributes to tumor early recurrence. Theoretically, treatment of these minimal intrahepatic metastases plays an important role in preventing of early recurrence of HCC. For these patients, postoperative adjuvant TACE had a role in earlier therapy of the residual tumor and could decrease the earlier recurrence and prolong survival. However, TACE has been known to damage remnant liver and deteriorate liver function. This adverse impact is possible to affect long survival of patients with low-RMP expression who has no metastasis.

In conclusion, we identified RMP serve as a valuable prognostic biomarker with hepatocellular carcinoma. Patients with high-RMP expression were distinct from those with low expression and were associated with reduced survival but had a favorable response to adjuvant postoperative TACE. However, the “suggested” value for adjuvant TACE still needed to be addressed in future large-scale prospective trails.

## METHODS

### Patients and Tissue samples

We recruited 278 patients with HCC to a training cohort, from the Eastern Hepatobiliary Surgery Hospital, Second Military Medical University, Shanghai, China, from January, 2003, to January, 2005. Patients following the inclusion also had available paraffin embedded tumor tissues underwent tissue microarray (TMA) analysis: preoperative World Health Organization performance status of 0-1; Child-Pugh class A; no distant metastases, visualizable ascites, or encephalopathy; no chemotherapy or radiotherapy before surgery; curative resection; and resected lesions identified as HCC on pathological examination. 15 patients were excluded because of hepatic angiography after the operation indicating tumor straining and therapeutic TACE was performed. Curative resection of HCC was performed as described [29]. An independent cohort of consecutive 270 patients from July, 2006, to January, 2007 was collected. Of these, 247 met the same inclusion and exclusion criteria. These patients formed the validation cohort of this study. The study was approved by the institutional ethics committee. Informed consent was obtained before surgery.

### Adjuvant TACE

Adjuvant TACE mentioned in this article was preventive TACE and performed 1-2 months after hepatectomy. Hepatic arterial angiography was performed firstly. Among the patients without tumor stain in the remnant liver, preventive chemoembolization was done. The regimen for preventive adjuvant TACE consisted of 5-fluorouracil (5-FU) 0.75g, cisplatin (DDP) 60 mg, and the emulsion mixed with mitomycin C (MMC) 16 mg and lipiodol 5 ml. In the patients with tumor stain, therapeutic TACE was performed according to the tumor size and number, and this is not the adjuvant preventive TACE mentioned in this article. One month later a Contrast-enhanced CT or magnetic resonance imaging was performed and the regimen was finished.

### Follow Up

Patients were observed once every 2 months in the first 2 years after surgery and then every 3 to 6 months thereafter. At each of the follow-up visits, a detailed history and a complete physical examination were carried out. Blood was taken for serum AFP, and liver function tests, and an abdominal ultrasound was carried out. Contrast-enhanced CT or magnetic resonance imaging was performed once every 6 months or earlier when tumor recurrence or metastasis was suspected.

Patients with intrahepatic or extrahepatic recurrences were treated with surgery, local ablative therapy, regional therapy, or systemic therapy, depending on the size, location and number of recurrent tumors, liver function status. Palliative treatment was given to patient with advanced disease, poor liver function or poor general status. Overall survival (OS) was defined as the interval between partial hepatectomy and death or the last date of follow-up. Disease free survival (DFS) was defined as the interval between hepatectomy and recurrence or last date of follow-up.

### TMA and Immunohistochemical Analysis

After screening hematoxylin and eosin-stained slides for optimal tumor content, we constructed TMA slides (Shanghai Biochip Company, Ltd., Shanghai, China). Immunohistochemistry was performed as described before [30]. The sections were incubated in the primary polyclonal antibodies against RMP (Proteintech, USA). Stained sections were evaluated in a blinded manner without prior knowledge of the clinical information using the German immunoreactive score, immunoreactive score (IRS) as described before [31]. Briefly, the IRS assigns sub-scores for immunoreactive distribution (0–4) and intensity (0–3), then multiplies them to yield the IRS score. The percent positivity was scored as “0” (<5%), “1” (5–25%), “2” (25–50%), “3” (50–75%) or “4” (>75%). The staining intensity was score as “0” (no staining), “1” (weakly stained), “2” (moderately stained) or “3” (strongly stained). Cases with discrepancies in IRS score were discussed together with other pathologists until consensus was reached.

### Statistical Analysis

OS or DFS was considered the primary endpoints. OS was calculated from the date of resection to the date of death or last follow-up. DFS was calculated from the date of resection to the date of recurrence or last follow-up. All statistical analyses were performed with SPSS version 18.0 software. The Fisher's exact test was used to compare qualitative variable. Survival curves were calculated using the Kaplan-Meier method and compared using a log-rank test. The Cox proportional hazards model was used to determine the independent factors on survival and recurrence, based on the variables selected on univariate analysis. P<0.05 was considered statistically significant.

## SUPPLEMENTARY FIGURES AND TABLES


