# Supplementary material for: RMP predicts survival and adjuvant TACE response in hepatocellular carcinoma
Source: Oncotarget. 2014 Dec 30;6(5):3432–42. doi: 10.18632/oncotarget.3092 (PMC4413664; doi:10.18632/oncotarget.3092)
Supplement: Supplementary file 1 [file oncotarget-06-3432-s001.pdf]

RMP predicts survival and adjuvant TACE response in hepatocellular carcinoma

Supplementary Material

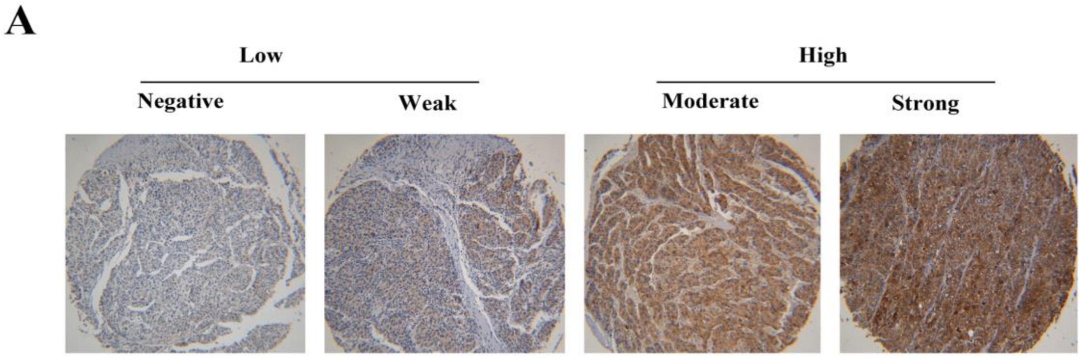

Figure S1: Immunohistochemical Analysis of HCC patients.

(A) Respective image of RMP in HCC patients.

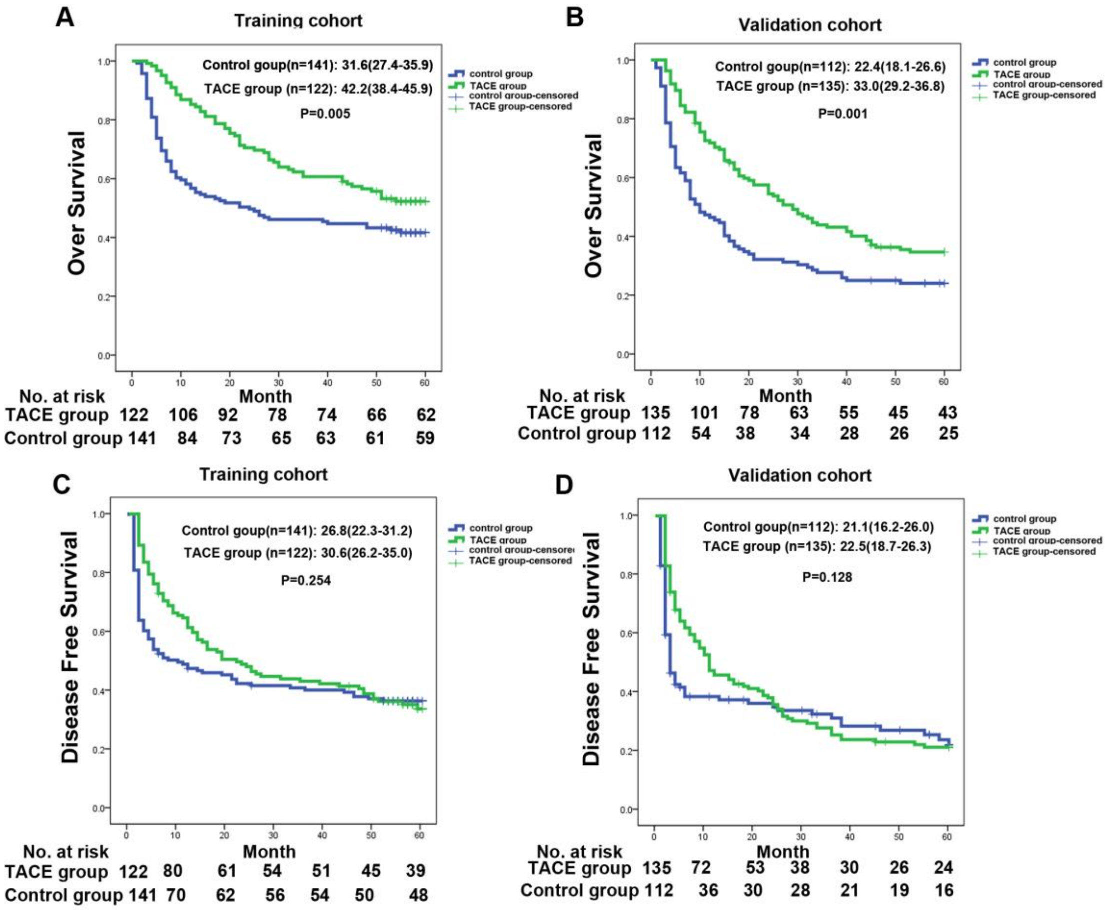

Figure S2: Prognostic significance of postoperative adjuvant TACE.

(A,B) Kaplan-Meier analysis of the correlation between adjuvant TACE therapy and OS in training cohort (A) and validation cohort (B). (C,D) Kaplan-Meier analysis of the correlation between adjuvant TACE therapy and 5 years disease free survival in training cohort (C) and validation cohort (D).

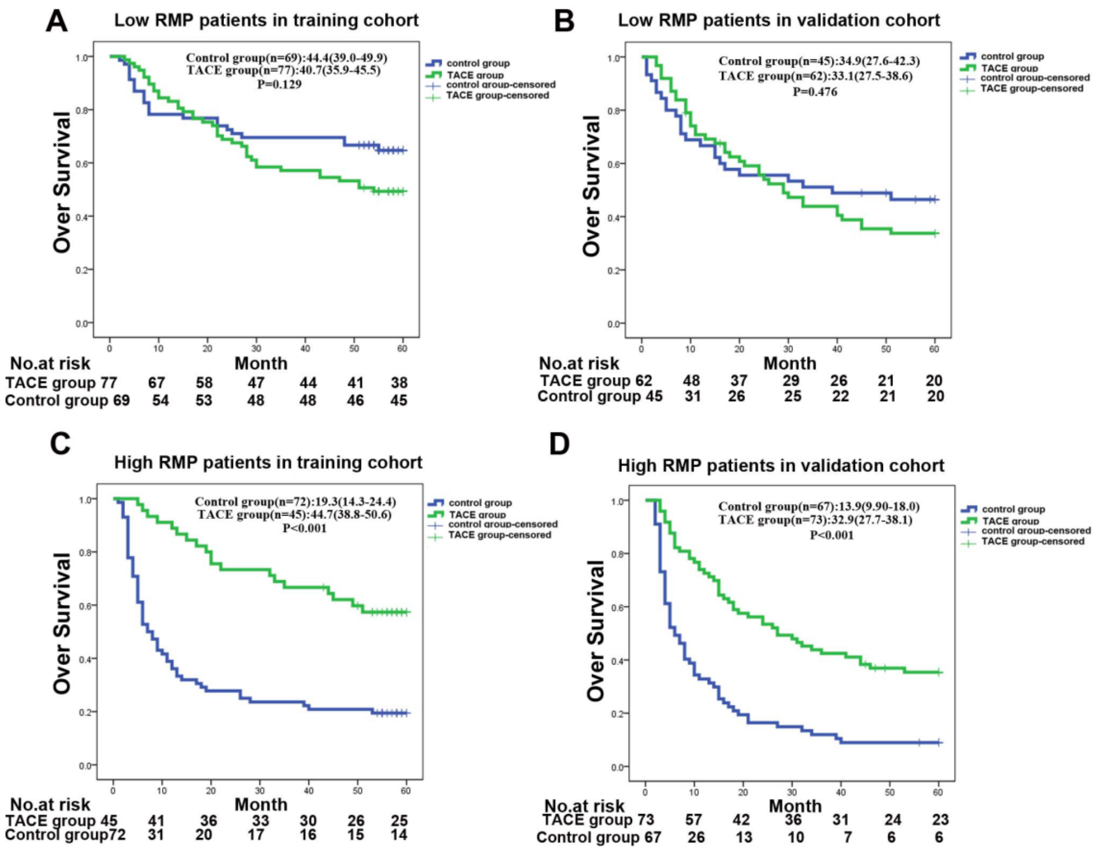

Figure S3: Prognostic significance of postoperative adjuvant TACE within the RMP level.

(A,B) Kaplan-Meier analysis of the correlation between adjuvant TACE therapy and OS in patients with low RMP expression in training cohort (A) and validation cohort (B). (C,D)Kaplan-Meier analysis of the correlation between adjuvant TACE therapy and OS in patients with high RMP expression in training cohort (C) and validation cohort (D).

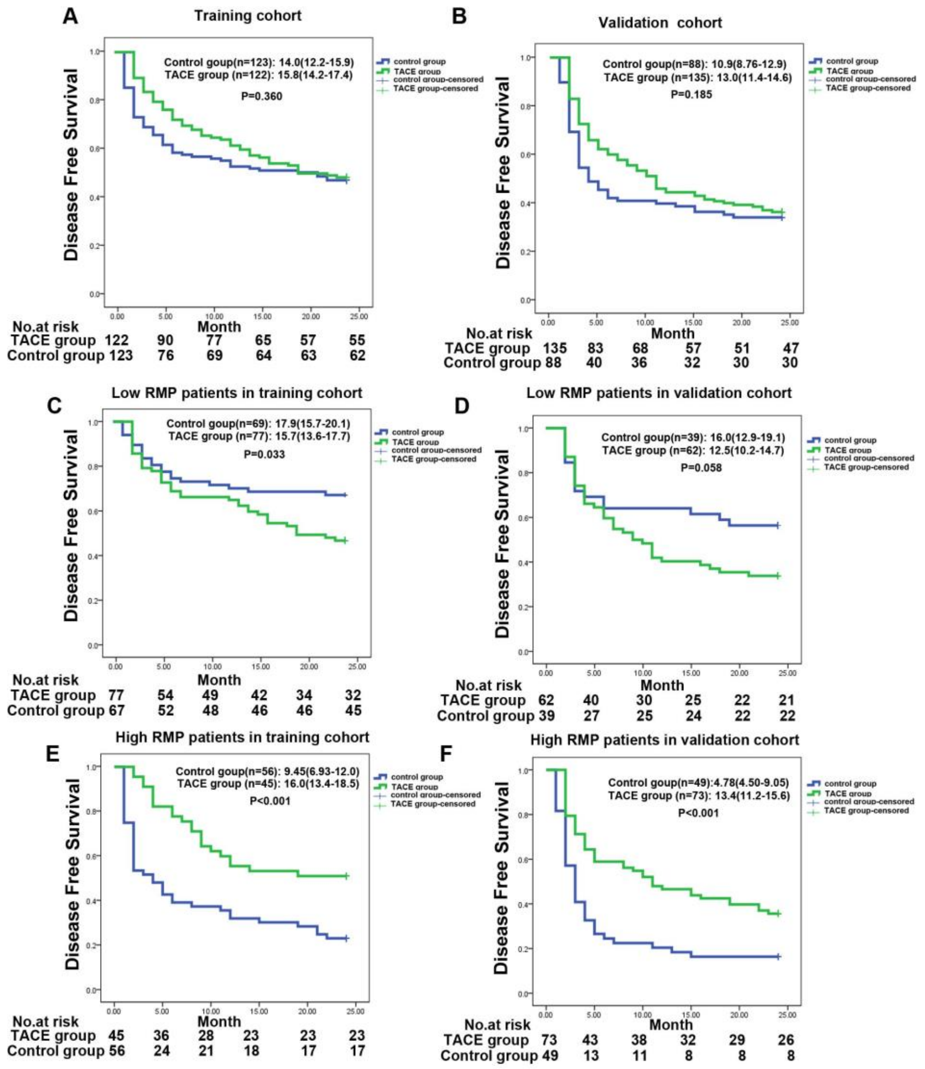

Figure S4: Prognostic significance of postoperative adjuvant TACE within the RMP level.

(A,B) Kaplan-Meier analysis of the correlation between adjuvant TACE therapy and 2 years disease free survival in training cohort (A) and validation cohort (B). (C,D) Kaplan-Meier analysis of the correlation between adjuvant TACE therapy and 2 years disease free survival in patients with low RMP expression in training cohort (C) and validation cohort (D). (E,F) Kaplan-Meier analysis of the correlation between adjuvant TACE therapy and 2 years disease free survival in patients with high RMP expression in training cohort (E) and validation cohort (F). All above results were analysis excluding the patients who were bad performance status and poor liver function after resection.

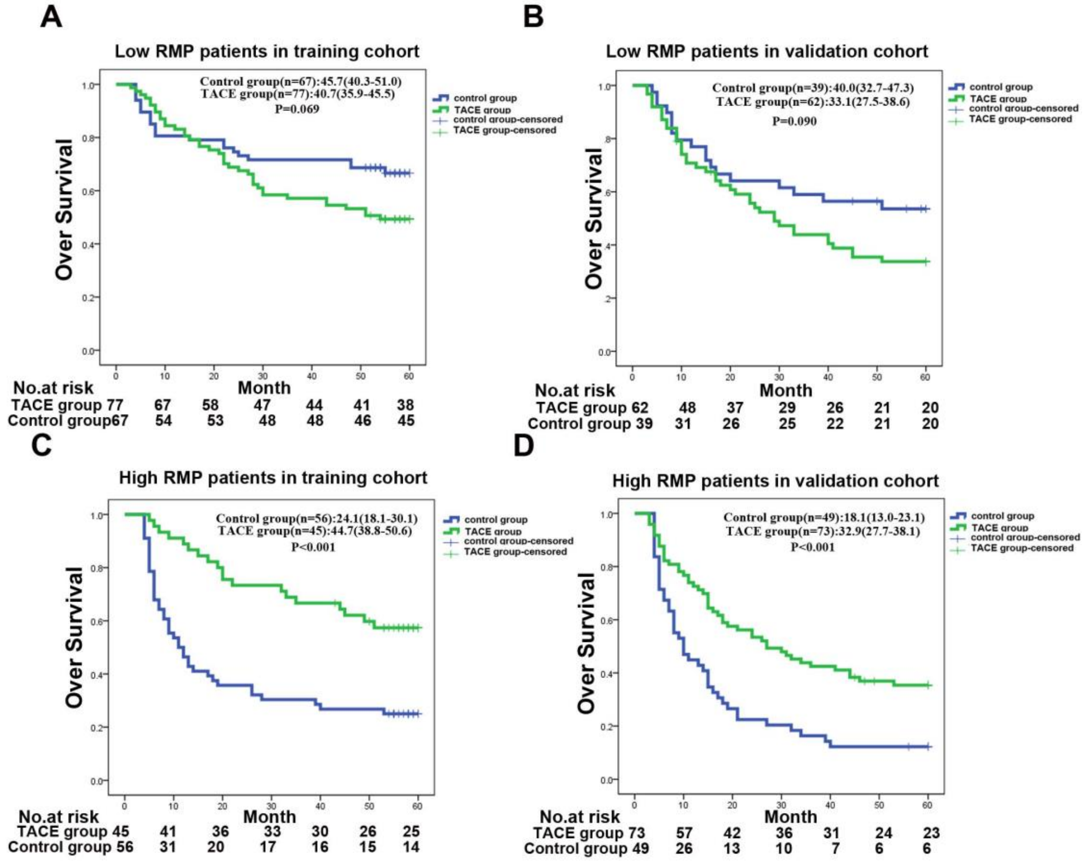

Figure S5: Prognostic significance of postoperative adjuvant TACE within the RMP level.

(A,B) Kaplan-Meier analysis of the correlation between adjuvant TACE therapy and OS in patients with low RMP expression in adjust training cohort (A) and adjust validation cohort (B). (C,D)Kaplan-Meier analysis of the correlation between adjuvant TACE therapy and OS in patients with high RMP expression in adjust training cohort (C) and adjust validation cohort (D).

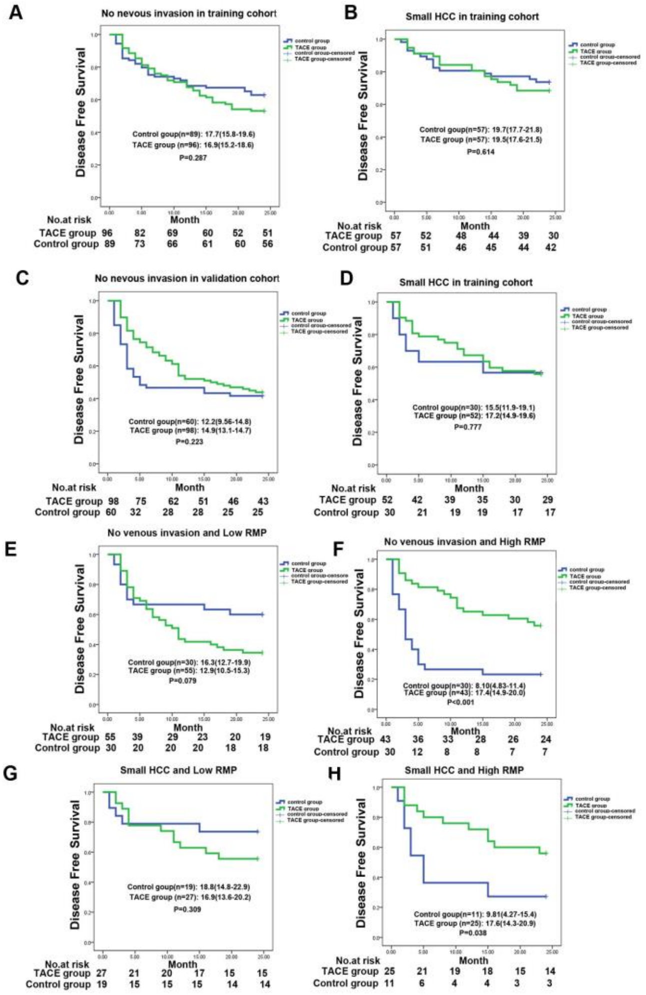

Figure S6: RMP predicts response to postoperative TACE in several clinical subgroups.

(A,B) Kaplan-Meier analysis of the correlation between adjuvant TACE therapy and 2 years disease free survival in patients with no venous invasion(A) or small HCC(B) in training cohort. (C,D) Kaplan-Meier analysis of the correlation between adjuvant TACE therapy and 2 years disease free survival in patients with no venous invasion(A) or small HCC(B) in validation cohort. (E,F) Kaplan-Meier analysis of the correlation between adjuvant TACE therapy and 2 years disease free survival in patients with no venous invasion at different RMP expression in validation cohort, low RMP(E) and high RMP (F). (G,H) Kaplan-Meier analysis of the correlation between adjuvant TACE therapy and 2 years disease free survival in patients with small HCC at different RMP expression in validation cohort, low RMP(G) and high RMP (H).

Table S1: Relationship between RMP protein expression and clinicopathologic characteristics in validation set (n=247)

Characteristics	No. patients	RMP expression in HCC		P value
		Low	High	
Age (yrs)				**0.000**
≤49	82	20	62	
＞49	165	87	78	
Gender				**0.002**
Male	213	84	129	
Female	34	23	11	
HBs Ag				0.669
Negative	35	14	21	
Positive	212	93	119	
Serum AFP				**0.004**
≤400（ng/ml)	82	46	36	
＞400（ng/ml)	165	61	104	
Largest tumor size				**0.004**
≤5 (cm)	82	46	36	
＞5 (cm)	165	61	104	
Tumor number				0.584
Single	209	89	120	
Multiple	38	18	20	
Venous invasion				**0.000**
Negative	158	85	73	
Positive	89	22	67	
BCLC stage				**0.000**
A	75	49	26	
B	83	37	46	
C	89	21	68	
TNM				**0.000**
I/ II	126	69	57	
III/IV	121	38	83	

Table S2: Univariate and multivariate Cox regression analyses of 2 years DFS in different RMP expression patients of the validation cohort.

	Low-RMP		High-RMP	
**Variables**	**Hazard ration (95% CI)***	**p Value**	**Hazard ration (95% CI)***	**p Value**
**Univariate analysis**				
adjuvant TACE (yes vs no)	1.256(0.753-2.097)	0.382	0.412 (0.278-0.609)	**0.000**
Age (>49 years vs ≤49 years)	0.733 (0.405-1.326)	0.304	0.524(0.356-0.771)	**0.000**
Gender (male vs female)	1.651(0.946-2.882)	0.078	1.603(0.833-3.083)	0.158
HBs Ag (negative vs positive)	2.393 (0.868-6.593)	0.092	1.354(0.784-2.339)	0.277
Serum AFP (>400 ng/ml vs≤400 ng/ml)	1.198 (0.722-1.987)	0.485	1.777 (1.145-2.758)	**0.010**
Largest tumor size (>5 cm vs ≤5 cm)	3.017(1.722-5.287)	**0.000**	2.109(1.303-3.414)	**0.001**
Tumor number(single vs multiple)	0.832(0.424-1.634)	0.594	0.751(0.420-1.345)	0.336
Venous invasion (negative vs positive)	1.786(1.012-3.154)	**0.045**	2.632(1.765-3.926)	**0.000**
BCLC stage (A vs B vs C)	1.598(1.172-20177)	**0.003**	2.086(1.563-2.784)	**0.000**
TNM (Ⅰ+Ⅱ vs Ⅲ+Ⅳ)	1.710(1.039-2.814)	**0.035**	2.887(1.885-4.422)	**0.000**
Multivariate analysis				
adjuvant TACE (yes vs no)	NA		0.434 (0.291-0.646)	**0.000**
Age (>49 years vs ≤49 years)	NA		NA	
HBs Ag (negative vs positive)	NA		NA	
Serum AFP (>400 ng/ml vs≤400 ng/ml)	NA		1.657(1.061-2.586)	**0.026**
Largest tumor size (>5 cm vs ≤5 cm)	3.017(1.722-5.287)	**0.000**	NA	
Venous invasion (negative vs positive)	NA		NA	
BCLC stage (A vs B vs C)	NA		1.938(1.448-2.593)	**0.000**
TNM (Ⅰ+Ⅱ vs Ⅲ+Ⅳ)	NA		NA	

Univariate analysis, Cox proportional hazards regression; Multivariate analysis, Cox proportional hazards regression; Variables were adopted in multivariate analysis for their prognostic significance by univariate analysis.
